# Multiorgan failure in a young patient with necrotizing fasciitis: a case report of septic shock progressing to mixed shock with fatal cardiac outcome

**DOI:** 10.1186/s13256-026-06805-2

**Published:** 2026-11-13

**Authors:** Richard Felix Kraus, Christina Kaufmann, Volker Alt, Martin Georg Kees

**Affiliations:** 1 Department of Anaesthesiology and Intensive Care Medicine, University Hospital Regensburg, Franz‐Josef‐Strauss‐Allee 11, 93053 Regensburg, Germany; 2 Department for Trauma Surgery, University Hospital Regensburg, Franz‐Josef‐Strauss‐Allee 11, 93053 Regensburg, Germany

**Keywords:** Necrotizing fasciitis, TAVI, Infective endocarditis, Paravalvular abscess, Severe heart failure, Multidisciplinary management

## Abstract

**Background:**

Necrotizing soft tissue infections (NSTIs) are rare, life-threatening conditions requiring immediate and aggressive treatment. Despite advances in medical care, mortality remains high, largely due to septic shock and severe complications.

**Case representation:**

We report the case of a young adult with necrotizing fasciitis (NF) of the left leg complicated by recurrent gastrointestinal bleeding and infective endocarditis, resulting in severe heart failure and ultimately leading to death. The fatal outcome was most likely attributable to prolonged sepsis with multiorgan involve- and impairment and a 1.5-cm paravalvular abscess caused by vancomycin-resistant Enterococcus faecium.

**Conclusion:**

The treatment of a septic patient with progressive heart failure highlights the value of an integrative, multidisciplinary approach to complex clinical decision-making and the real-world challenges that may require patient-tailored, nonstandard therapeutic strategies. Prompt diagnosis, aggressive surgical debridement, and rigorous management of complications are essential. In cases of aortic valve regurgitation causing heart failure, TAVI implantation might serve as a temporary bridge until definitive open valve replacement becomes feasible.

**Supplementary Information:**

The online version contains supplementary material available at https://doi.org/10.1186/s13256-026-06805-2.

## Introduction

Despite being a rare condition (global incidence is reported as 0.4/100,000 per year), necrotizing soft tissue infections (NSTIs) are extremely lethal conditions requiring immediate and aggressive intervention for resuscitation and to limit mutilation and loss of limbs [1]. Despite the improvements in our understanding and medical care the mortality rate of NSTIs has not changed in the past 30 years and is estimated at 6 to 35 % [1, 2]. NSTIs typically lead to septic shock with or without bacteremia. Thereby, mortality is not limited to the early phase of disease but also determined by subsequent, severe complications of infectious and non-infectious nature, such as infective endocarditis, secondary wound infections, renal failure and gastroduodenal ulcers [3]. Whereas standardized approaches for each of these conditions exist when they present alone, matters become much more complicated and may require unconventional or preliminary solutions when they coincide in a critically ill patient. An experienced multidisciplinary team should be available for swift and sound decision-making [4].

**Fig. 1:**
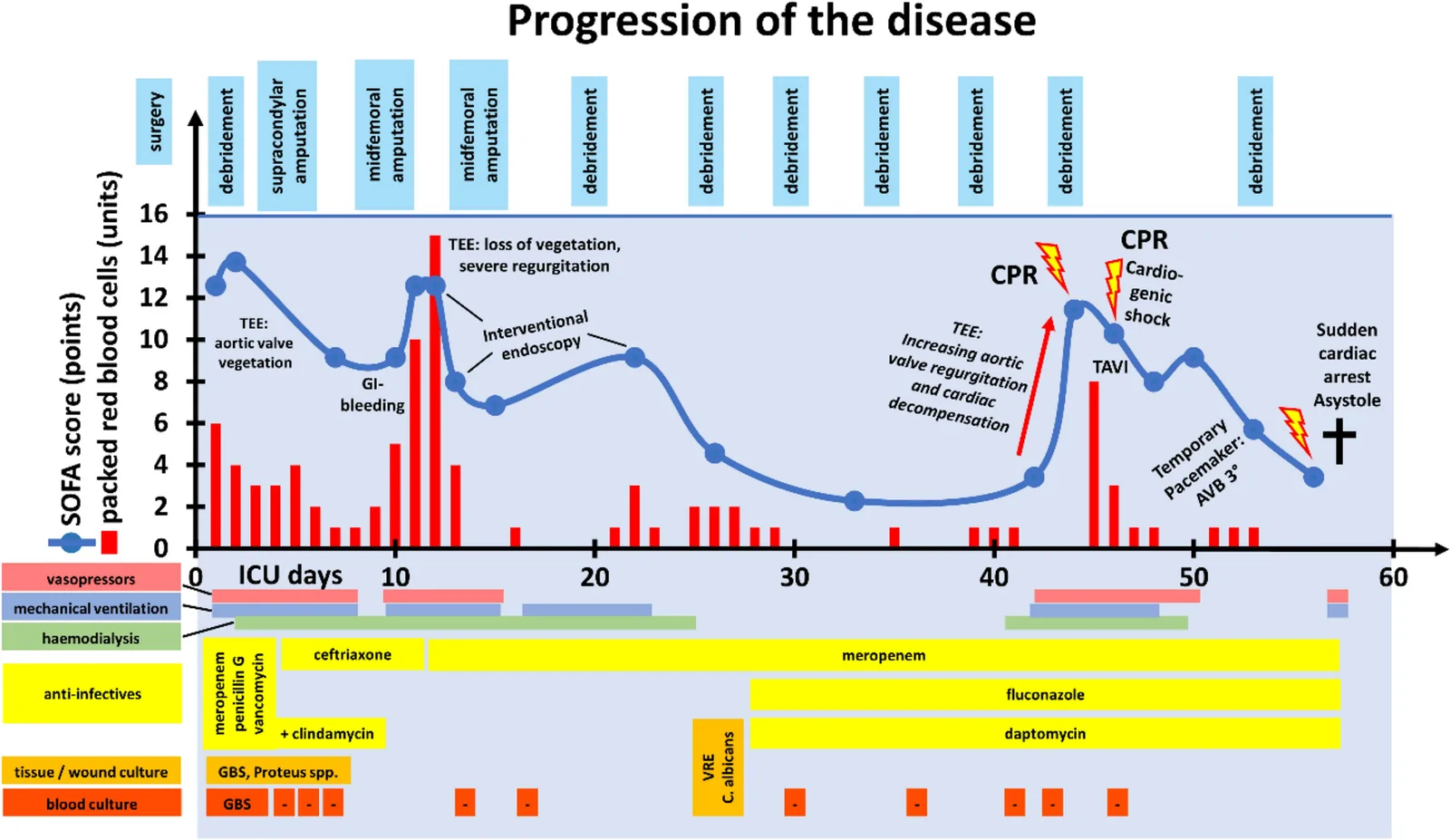
Schematic course of the disease with pivotal therapeutic interventions. Organ dysfunctions are indicated by the SOFA score. *GBS* group B streptococci, *ICU* intensive care unit, *VRE* vancomycin resistant enterococcus, *C. albicans* candida albicans, *TEE* transesophageal echocardiography, *CPR* cardiopulmonary resuscitation, *AVB* *3* atrioventricular block third degree, *TAVI* transcatheter aortic-valve implantation, *GI-bleeding* gastrointestinal bleeding

Here we report a case (timeline see Fig. 1) of a young adult with necrotizing fasciitis of the left leg complicated by recurrent gastrointestinal bleeding and infective endocarditis leading to heart failure and ultimately to death.

## Case

**Fig. 2:**
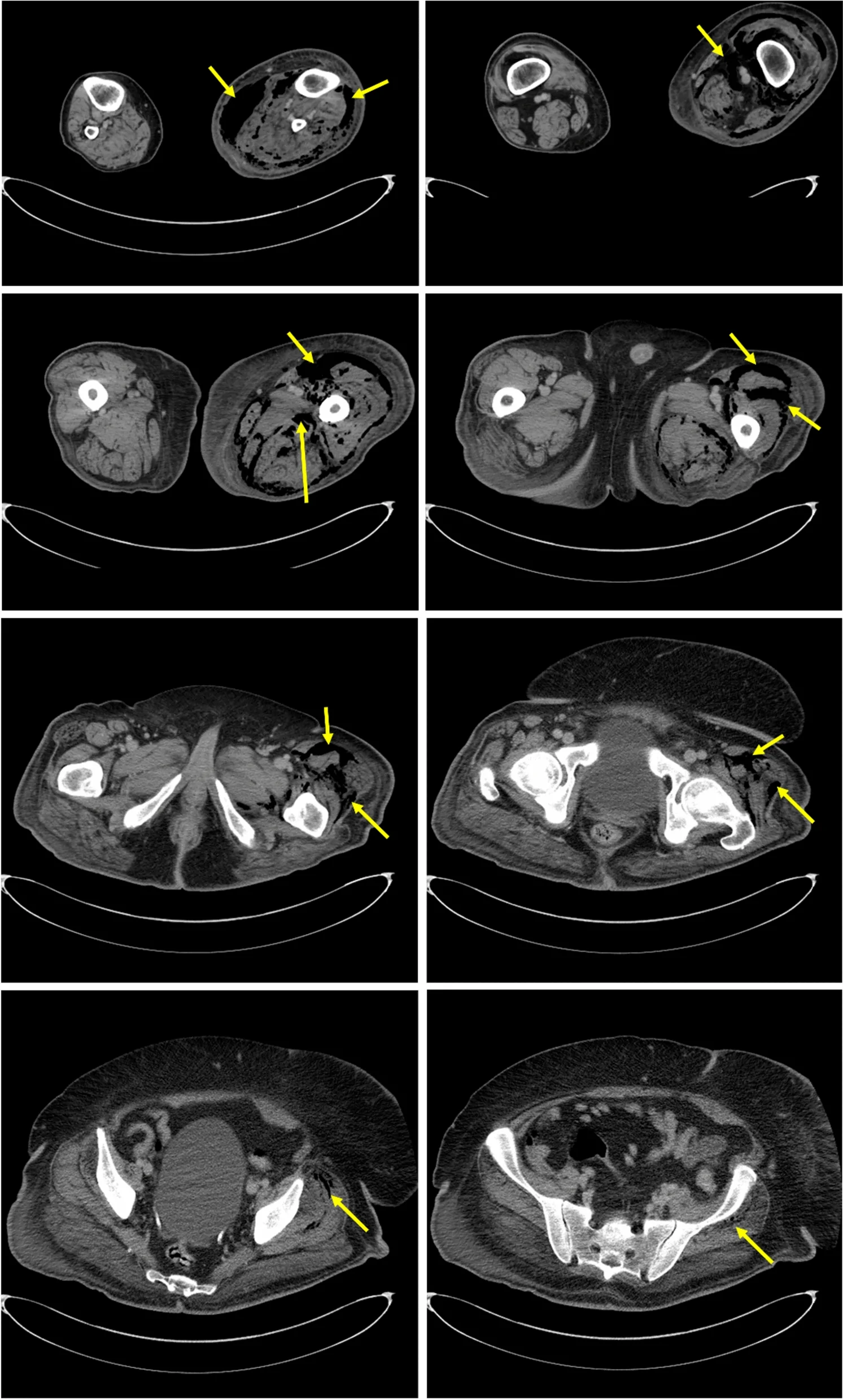
Initial CT graphic examination in our emergency department: Swollen presentation of the soft tissue of the entire left leg as well as diffuse air pockets. Starting distally on the left foot with osteodestruction of the tarsal bones as well as intraosseous air pockets in the talus and calcaneus (here not entirely shown). The changes extend further proximally into the left gluteal musculature. No evidence of osteodestruction of the lower leg bones, the femur or the bony pelvis. No evidence of a fracture in the scan area. Highly suspected necrotizing fasciitis of the entire left leg, proximal extension into the gluteal muscles. Extensive osteodestruction of the left foot due to the inflammatory changes

A white man in his late thirties had had several purulent lesions on his left lower leg for months, for which he had refused to seek professional medical expertise. Instead, he immobilized the leg in a splint and presumably took non-steroidal anti-inflammatory drugs for a longer period. He denied any trauma or bite in this location. Two months before admission, Type II Diabetes Mellitus had been diagnosed. Eventually, his general condition had deteriorated significantly over the past few days, so he was admitted by the emergency medical service to our hospital. Initial examination in our emergency department showed necrotizing fasciitis of the left lower extremity extending upwards into the thigh (Fig. 2).

The LRINEC Score (Laboratory Risk Indicator for Necrotizing Fasciitis) resulted in 13 points, which supports high risk for NSTI (scores ≥ 8 had a PPV of 93.4 % for NSTIs) [5]. Empirical antibiotic therapy was started with meropenem, penicillin G, clindamycin and vancomycin (Fig. 1).

**Fig. 3:**
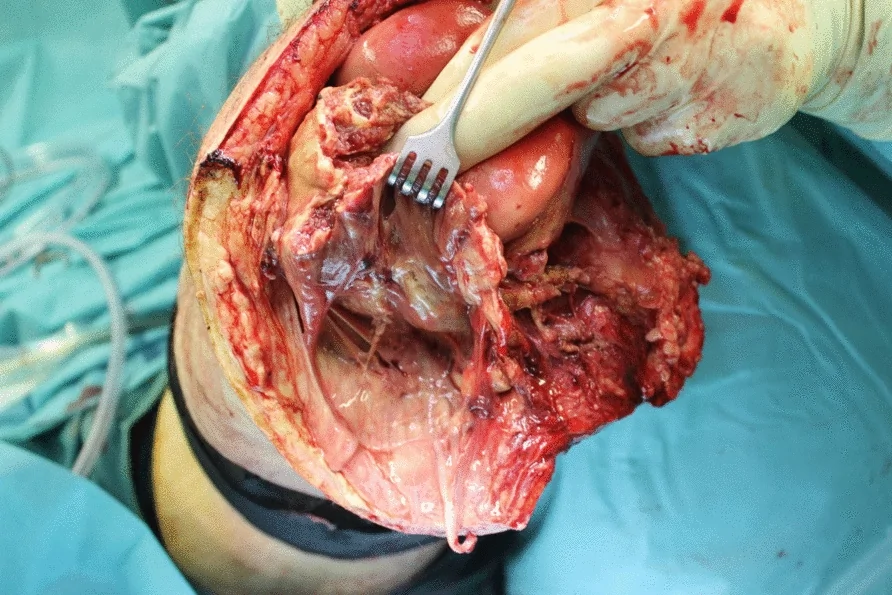
Progressing deep soft tissue infection extending to the bone, making dysarticulation of the knee and consecutive distal femoral amputation necessary

The patient was immediately taken to the operating theater for surgical exploration and extensive debridement (Fig. 3). The operative procedure entailed extensive surgical debridement of the left limb with lateral skin incisions over the thigh to access all fascial compartments. Infected tissues were excised, and multiple tissue samples were obtained for microbiological and histological examination. The procedure included fascial releases, extensive jet lavage, and the application of hypochlorous acid (GranuDacyn) for chemical debridement. The knee was opened with an arthrotomy and a synovectomy was performed. The wounds were temporarily closed with an artificial wound coverage (Epigard®; Biovision, Ilmenau) and compression bandages were applied.

Despite the severe affection, a limb-preserving approach was applied because musculature seemed vital and drainage of pus seemed sufficient. Postoperatively he was transferred to our operative intensive care unit.

The first days were characterized by moderate transfusion requirements and relative circulatory instability due to diffuse bleeding from the surgical site. Haemodialysis was started for acute renal failure with severe uremia (> 400 mg/dl). The first revision operation on day 2 revealed deep soft tissue infections extending to the bone, making distal femoral amputation necessary.

Abundant growth of Group B streptococci (GBS) was reported on day 2 in tissue samples and in blood cultures; additionally, superficial wound swabs showed some growth of Proteus species. Antibiotic therapy was de-escalated to ceftriaxone and clindamycin. A transesophageal echocardiography performed on day 2 also revealed the involvement of the aortic valve, which initially appeared competent but with a very large vegetation and corresponding risk of embolism (Fig. 6 (a+b) and Video 1 in the supplement). In addition, bleeding of the upper gastrointestinal tract occurred due to esophagitis and a large duodenal ulcer (most likely after prolonged Ibuprofen intake). Thus, open surgery for aortic valve replacement requiring full heparinization for cardiopulmonary bypass did not seem possible at this time.

The situation improved over the next few days so that the patient could be extubated and catecholamine therapy could be temporarily discontinued.

On day 5, a colonoscopy showed some minor ulcers in the distal rectum caused by the fecal management catheter, which was removed. The patient was intubated again on day 10 because of recurring gastrointestinal hemorrhage and pending revision surgery of the leg. Esophagogastroduodenoscopy and coloscopy were performed and showed abundant old blood in the entire colon, but no active hemorrhage at this time. The intraoperative findings required an extension of the femoral amputation and the initiation of a vacuum assisted closure-therapy (VAC-therapy). Due to the dubious wound conditions, we escalated the antibiotic therapy to meropenem (Fig. 1).

**Fig. 4:**
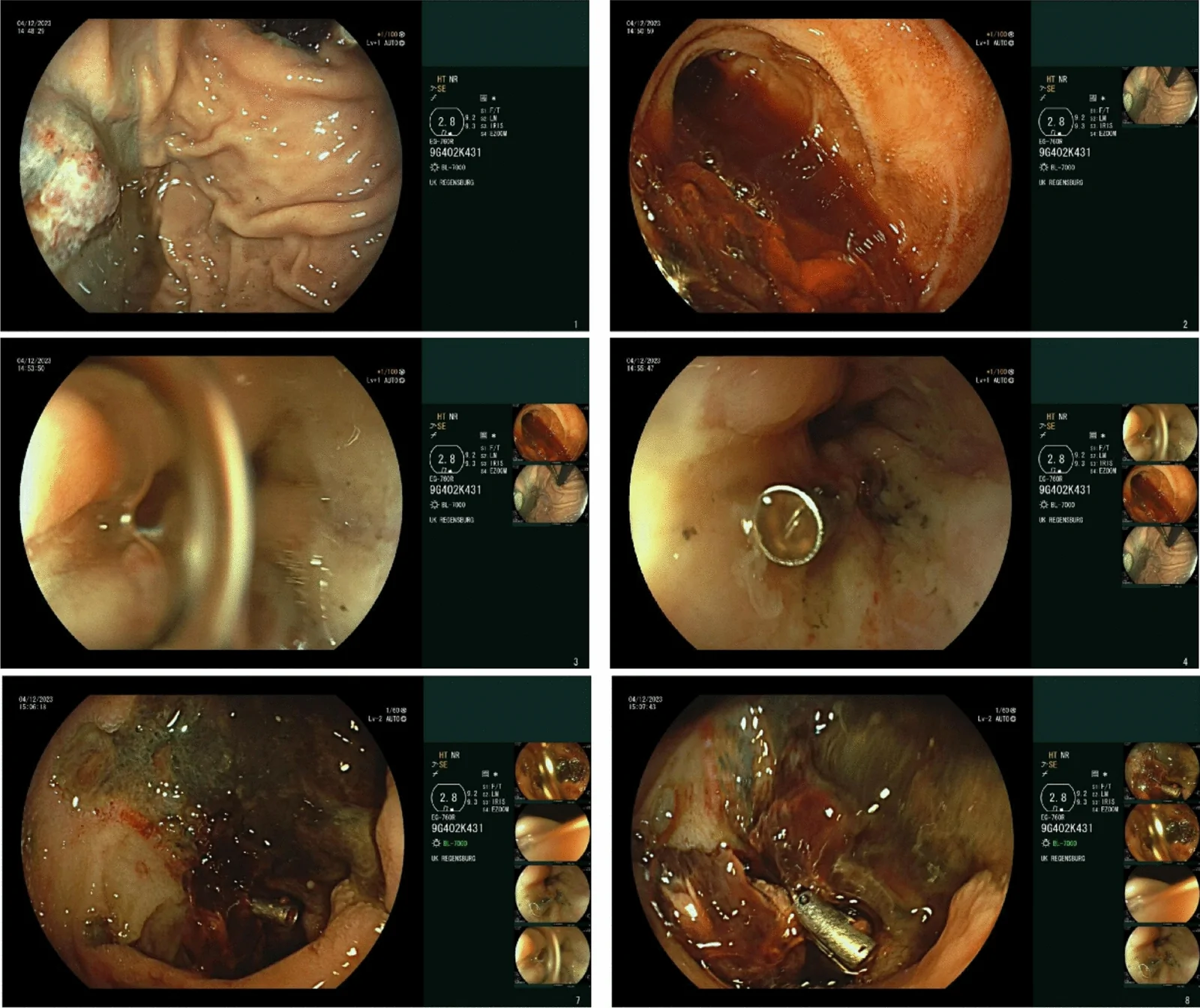
Gastroduodenal bleeding was stopped endoscopically by over-the-scope clipping and cryoablation

During the night from day 11 to 12 (shortly after the second revision operation), a severe gastrointestinal hemorrhage with hemorrhagic shock occurred, necessitating a massive transfusion of blood products. Finally, the source of the hemorrhage in the duodenum was detected during gastroscopy, treated with clipping and cryoablation (Fig. 4).

**Fig. 5:**
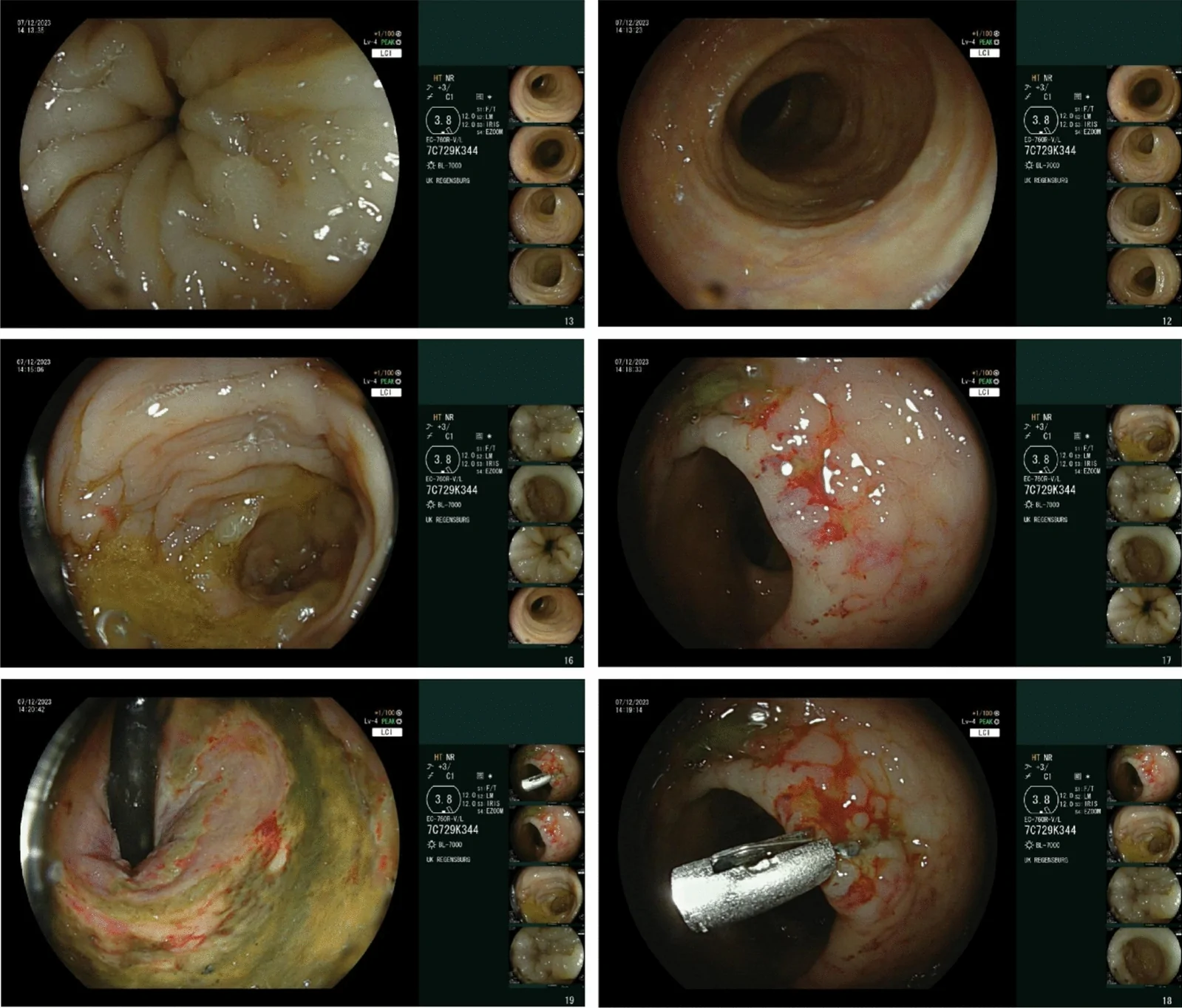
Intestinal bleeding from the lower gastrointestinal tract had also to be treated with clipping

After rapid circulatory stabilization, another severe gastrointestinal bleeding event and corresponding massive transfusion occurred again the following night. The gastroduodenal artery was then closed by angiographic coiling, and the bleeding stopped. On day 13, however, a new hemorrhage of the lower gastrointestinal tract had to be stopped endoscopically using clipping and haemospray (Fig. 5).

Afterwards the patient showed circulatory stability under moderate catecholamine therapy and could be extubated again on day 15 after a further shortening of the amputation stump. After extubation, the vigilance and speech production improved, but we detected a proximally accentuated paresis of the left arm.

**Fig. 6:**
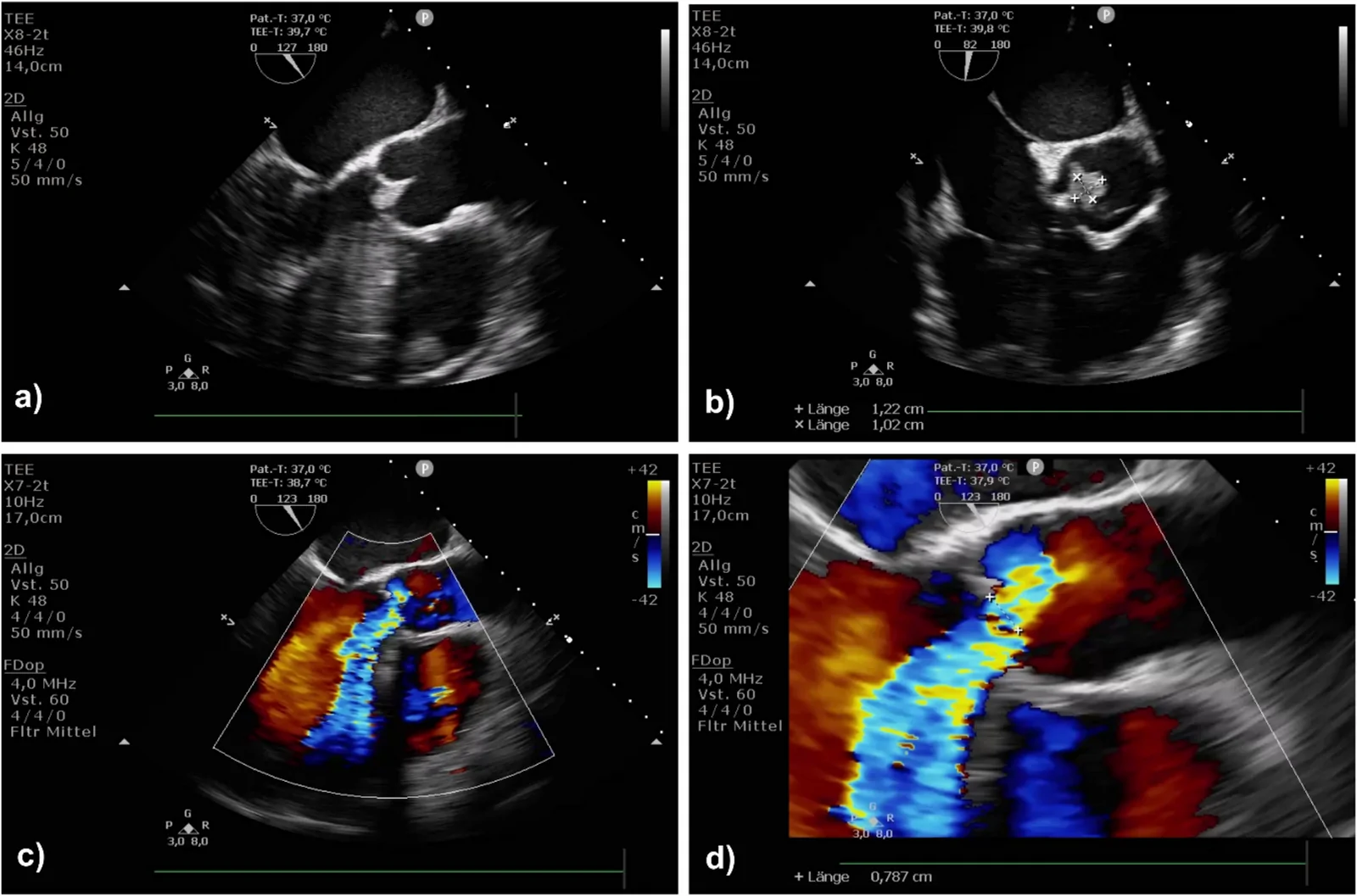
Transesophageal echocardiographic assessment of infective endocarditis: visualization of an aortic valve vegetation (on Day 2: (a+b)) and subsequent aortic regurgitation following vegetation detachment (on Day 15 (c+d)). **a** Mid-esophageal long-axis TEE view at 127° demonstrating a vegetation attached to the aortic valve. **b** Mid-esophageal short-axis TEE view at 82° demonstrating the aortic valve vegetation, measuring 1.22 × 1.02 cm. **c** Color Doppler TEE view at 123° demonstrating aortic regurgitation following detachment of the vegetation. **d** Magnified color Doppler view in the same plane showing the regurgitant jet, with a measured jet width of 0.79 cm

In the echocardiographic control on day 15, the vegetation was no longer present and the aortic valve was destroyed with grade IV regurgitation (Fig. 6 (c+d) and Video 2 in the supplement), so a cardioembolic insult was assumed. The cranial computer tomography showed an ischemic correlate in the right precentral gyrus.

Over the following days, catecholamine support could be reduced to a minimum, while blood transfusions were only rarely required. Except for the need for further surgical re-resections, mechanical ventilation could be avoided. In the setting of negative blood cultures and an overall improving clinical condition, we decided to continue the established treatment regimen with meropenem given the favorable clinical response (Fig. 1).

Despite extensive ulcer prophylaxis (for details see Discussion Sect. 3), on day 22 further hemorrhage from the duodenal ulcer requiring transfusion with hemodynamic instability occurred and lasted active for another 3 days, despite attempted angiographic embolization. Angiographic occlusion of the feeding arteries was unsuccessful. Thus, several “through the scope” (TTS) clips, injections with adrenaline, haemospray and argon laser were applied. Several tests for Helicobacter pylori remained negative. From day 23 we used somatostatin and misoprostol from day 26 as adjunctives. From then on, a clear improvement in the clinical findings was observed in the follow-up endoscopies, and no further treatment-relevant hemorrhage occurred.

In the following days the patient's clinical condition improved significantly and continuously. He regained his orientation, was able to make controlled movements of the right arm and right leg, was able to eat, catecholamine therapy could be stopped and diuresis stabilised so that the dialysis could be paused (minimum creatinine 0.83 $\frac{mg}{dL}$). However, from day 26 onwards osteomyelitis caused by vancomycin-resistant Enterococcus faecium and Candida albicans was detected in the intraoperative samples of the femoral stump (subsequent amputation), which were treated systemically and topically by inserting “PerOssal” pellets with fluconazole and with daptomycin (Fig. 1). We also continued the broad-spectrum therapy with meropenem due to the problematic hygienic conditions caused by the location of the operation area in proximity of the anus, which necessitated multiple unplanned changes of the VAC dressing.

Over the following days, blood cultures turned negative again, the extent of organ dysfunction gradually decreased, and the need for blood transfusions was low. Given the favorable clinical response, we decided to continue the aforementioned treatment regimen, which resulted in further clinical improvement, reflected by a sustained decrease in the SOFA score (Fig. 1).

From day 36 onwards, renal function deteriorated noticeably again, which we interpreted as a sign of cardiac decompensation due to the aortic regurgitation. Therefore we repeated transthoracic echocardiography, which revealed mildly reduced systolic function of the dilated left ventricle, and severe aortic regurgitation associated with a highly mobile structure measuring approximately 9 mm. Moreover, on day 38, transesophageal echocardiography demonstrated prolapse and a flail non-coronary cusp with extensive destruction of all aortic valve cusps, resulting in torrential aortic regurgitation (Video 3 in the supplement).

On days 44 and 46 during further stump revisions, the patient briefly required cardiopulmonary resuscitation. Whereas he recovered quickly without neurological impairment and little lasting circulatory compromise after the first episode, he went into frank cardiogenic shock after the second one, with high doses of norepinephrine and epinephrine, lactate levels topping at 87 mg/dL. Mean arterial blood pressure was maintained at about 50–60 mmHg, but diastolic pressure could hardly be elevated above 30 mmHg.

An interdisciplinary Heart Team Consensus was reached to pursue urgent transcatheter aortic valve implantation (TAVI), as the patient´s condition (with still uncontrolled osteomyelitis and unresolved wound conditions) made open valve replacement on cardiopulmonary bypass prohibitively high risk**.** The main factors rendering conventional surgical valve replacement prohibitive were progressive haemodynamic deterioration, requiring mechanical cardiopulmonary resuscitation twice within 24 h, profound haemodynamic instability, dialysis dependence, and a EuroSCORE II of 77 % [6].

An interdisciplinary consensus was reached to pursue the urgent implantation of a transcatheter aortic-valve implantation (TAVI), since the patient´s condition (with still uncontrolled osteomyelitis and unresolved wound conditions) proved an open operation for valve replacement on cardiopulmonary bypass as too great a risk. Notably, despite the presence of a localized infection of the femoral stump, repeated blood cultures obtained the days prior to TAVI implantation remained negative (Fig. 1).

Therefore, preprocedural multidetector computed tomography (MDCT) was used for three-dimensional assessment of the aortic annulus and patient-specific TAVI planning and sizing, based primarily on the CT-derived annular area according to established sizing recommendations [7]. A 29 mm Edwards Sapien 3 transcatheter heart valve was selected. Preprocedural evaluation additionally revealed mild atherosclerotic changes with calcified plaques in the aortic arch, but no significant calcification at the aortic valve level.

The TAVI procedure was performed on day 46, 13 h after the second episode of cardiac arrest. According to our institution’s protocol (Video 4 and description in the supplement), a transjugular pacemaker was inserted for rapid pacing during the implantation and for the case of postinterventional atrioventricular blockade.

**Fig. 7:**
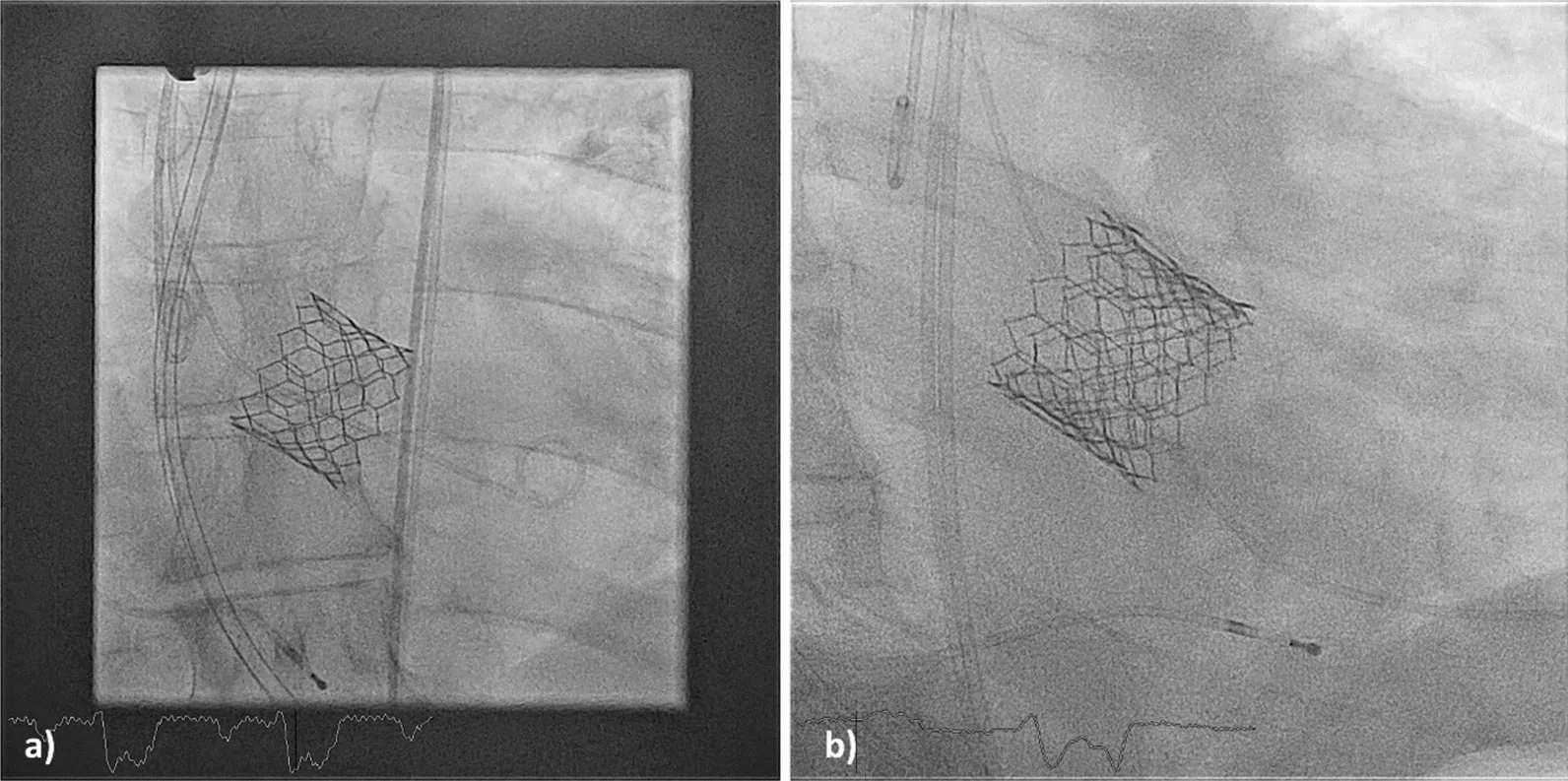
Sequential implantation of two 29-mm Edwards SAPIEN 3 transcatheter heart valves. **a** Fluoroscopic image after implantation of the first 29-mm SAPIEN 3 valve. **b** Fluoroscopic image following implantation of a second 29-mm SAPIEN 3 valve within the first prosthesis

Then, a 29 mm Edwards Sapien 3 transcatheter heart valve (Edwards Lifesciences Corporation, Irvine, USA) selected based on pre-procedural CT assessment, was implanted. Initial TEE confirmed good valve function; however, early hemodynamic assessment revealed migration of the prosthesis approximately 1 cm toward the LVOT with resulting supraskirtal aortic insufficiency, necessitating implantation of a second 29 mm Sapien 3 valve (same size) following re-crossing of the native valve (Fig. 7 and Video 4 in the supplement).

The evaluation of valve function, now performed using TEE, invasive hemodynamic measurement, and bulbography, showed normal opening characteristics of the prosthesis with only minimal regurgitation.

Following successful TAVI implantation, catecholamine requirements decreased, with epinephrine reduced from 0.6 mg/h to 0.3 mg/h and norepinephrine from 0.3 mg/h to 0.1 mg/h, while the vasopressin dose remained unchanged at 1 IU/h. Postoperatively, the patient was transferred to the ICU again for further care.

Due to the intense anticoagulation, there was still considerable bleeding from the stump requiring transfusion during the first night after the intervention, but this stopped by the following day. The circulatory insufficiency then resolved rapidly, so the patient could be extubated and weaned from catecholamines by day 48. In the absence of atrioventricular block, the transjugular pacemaker was removed on day 50, but inserted again on day 53, when a complete atrioventricular block occurred during a further revision operation on the femoral stump. Subsequently, the patient showed complete pacemaker dependency without the onset of a relevant ventricular replacement rhythm.

On day 56, the patient was in relative well-being and about to have lunch, when his invasively monitored blood pressure dropped suddenly from 135/60 to 30/10 mmHg, Peripheral oxygen saturation was at 98 %, and the ECG showed that the ventricle was correctly paced with a rhythm of 100/min (VVI). Pharmacological and then mechanical resuscitation were promptly initiated. The patient was intubated again. Echocardiography showed no cardiac activity. After about 15 min, the pacemaker was no longer able to stimulate the ventricle, and the ECG showed asystole. The pacemaker probe was repositioned during mechanical resuscitation, but without success. Transcutaneous pacemaking also failed. Use of extracorporeal life-support was discussed with a second senior intensivist, but was finally discarded because of perceived futility due to the still active osteomyelitis and the already existing sequelae of the long-lasting multiorgan dysfunction syndrome. Resuscitation efforts were therefore terminated after 30 min.

The autopsy revealed consequences of the long-lasting sepsis in liver, spleen and bone marrow. Dislocation of the aortic valve prosthesis, which had been clinically considered as the cause of the sudden circulatory failure, was excluded. There was a large paravalvular abscess of 1.5 cm diameter. Swabs of the prosthetic and the destroyed native valve showed growth of vancomycin-resistant Enterococcus faecium. Additionally, hypertrophy of the right ventricle, evidence of pulmonary emphysema and a thrombembolus of 1.2 cm in a peripheral branch of the pulmonary artery was found.

## Discussion

In this case, several per se life-threatening conditions coincided and ultimately led to the premature and tragic death of a young man. There are without doubt several crossroads where different decisions could have been taken, possibly with better outcomes:

1. Surgical approach – limb preservation vs. amputation:
2. Antibiotic and Fecal Management – prevention of wound contamination
3. Secondary prophylaxis of gastrointestinal bleeding
4. Timing and Modality of Aortic Valve Replacement in the Context of Antibiotic and Surgical Management of Endocarditis
5. Missed paravalvular abscess – secondary atrioventricular block might have prompted new TEE, possibly leading to salvage surgery
6. Decision and reasons not to initiate ECPR

### Ad 1: Surgical approach

Patients with suspected septic shock should be promptly managed in a critical care setting with heightened clinical vigilance and expedited diagnostics and treatment. Once the diagnosis of NF is suspected, immediate consultation with surgery is warranted [8]. While antibiotic therapy, resuscitation and critical care evaluation are necessary in the treatment of patients presenting with NF, the mainstay of the initial therapy remains surgical treatment with reduction of the bacterial load in the affected limb by surgical debridement and irrigation.

In definitely life-threatening conditions such as necrotizing fasciitis, attempts to save the limb may interfere with the chances to save life.

Multiple large studies have highlighted the importance of early and aggressive debridement in NF, and asserted it as the single most important treatment intervention for this disease process, although no randomized controlled trial has studied the timing or extent of surgical therapy or clearly defined an “adequate” debridement [8–11].

However, delay in the identification or delay in early surgical management clearly increases mortality [11]. In addition, recent data suggests that delay not only increases mortality, but in survivors, also increases the number of subsequent operations needed to control the infection [12].

Multiple operations can increase tissue loss, compromise functional recovery by sacrificing muscle and critical structures, raise costs, and thus highlight the importance of early diagnosis and aggressive treatment [8, 13, 14].

Although debridement is a cornerstone of treatment, no study has defined what an “adequate” debridement is, although typical training dictates that all necrotic tissue should be removed. Debridement, therefore, remains at the discretion of the operating surgeon [8, 15].

However, wounds should be re-evaluated in the operating room within 24–48 h after initial debridement to assess progression, remove necrotic tissue, and manage dressings, with an average of 3–4 procedures before transitioning to bedside care [8, 9, 12].

While vacuum-assisted closure devices are commonly used to manage soft tissue edema, support granulation, and simplify wound care, amputation was ultimately necessary due to larger scale debridements of entire muscle compartments, resulting in a nonfunctioning limb. In this context, the question arises whether an earlier, more radical amputation, in line with the “life before limb” principle, might have been considered. On reflection, a more radical surgical approach could potentially have contributed to better infection control; however, given the complexity of the clinical situation, it remains uncertain whether earlier surgical intervention would have altered the patient’s outcome. However, due to the heterogeneous appearance at the onset of the disease, it will always remain a case-by-case decision [8, 16–19].

Nevertheless, in our case, the extensive and complex debridements may have required multidisciplinary involvement—such as Urology for perineal and genital wounds. In addition, a loop colostomy may also have been required to protect the perineal wounds from secondary bacterial contamination from the colon (see Point 2) [8, 20].

### Ad 2: Antibiotic and fecal management

The necrotizing fasciitis was definitely caused by streptococci. Following an initial course of broad-spectrum antibiotic therapy, the treatment was adjusted based on resistance testing. Ceftriaxone was selected as targeted therapy because of its excellent activity against Streptococcus agalactiae, favorable safety profile, especially in the context of renal failure [21]. Clindamycin was added as adjunctive therapy to suppress streptococcal toxin production in the setting of necrotizing fasciitis. Evidence supporting this strategy in invasive Streptococcus agalactiae infection remains limited and is largely extrapolated from studies on group A streptococci, however, the isolated GBS strain was susceptible to clindamycin, further supporting its use as adjunctive therapy [22].

Later on, the wound and femur became infected with vancomycin-resistant enterococci and candida. Very probably this originated from contamination of the wound with feces. However, prompt and targeted antimicrobial therapy with fluconazole and daptomycin was initiated, likely contributing to the rapid clearance of these microorganisms from the bloodstream.

Of course, standard hygiene measures are crucial, but fecal contamination of a proximal amputation site of the femur is difficult to avoid completely. We temporarily used a commercial fecal management system (hygh-tec® basic-plus, Advanced Medical Balloons GmbH, Waghäusel, Germany) to collect liquid stool, but removed it because of bleeding from rectal ulcers caused by the sealing balloon. Surgical creation of a colostomy might have been a valid alternative. Even in Fournier’s gangrene with damage to the anal sphincter, there is no clear consensus that colostomy is beneficial. A systematic review and meta-analysis showed colostomy to be a predictor of poor outcome and high mortality, but only one among the 27 included studies had a prospective design [23]. A recent prospective, but non-randomized study found significantly shorter hospital stays and faster wound healing in 15 patients who underwent colostomy after the first debridement compared to 15 patients who did not consent to the procedure [24].

In the context of our case, the added morbidity of a temporary colostomy might have been negligible, and, in hindsight, might have been life saving.

### Ad 3: Management of gastrointestinal bleeding

Our approach to treating gastrointestinal hemorrhage at different sites throughout the gastrointestinal tract was according to clinical practice. Even before the first onset, we administered a prophylactic dose (20 mg o.d.) of esomeprazole from day 1, and established a high-dose intravenous administration of pantoprazole at the onset of bleeding on day 3 (days 3–5: 80 mg bolus following continuous application of 8 mg/h over 24 h and from day 6: 40 mg 4× daily). Interventional endoscopy was performed within hours and repeated several times in both the upper and the lower gastrointestinal tract, but did not prevent rebleeding. Interventional angiography also failed. We are not aware of clinical practice guidelines or recommendations for this case, probably because healing rates with PPI therapy are high [25]. Before PPI became available and dominant, the prostaglandin analogue misoprostol was commonly used and apparently of comparable efficacy to histamine-receptor-antagonists and in certain aspects even to PPI. Whereas PPI act by inhibition of acid secretion, the effect of misoprostol is suppored to be by “mucosal protection” [26, 27]. Therefore, the combination therapy of a PPI and might theoretically have complementary effects on mucosal protection and healing;. Still, the potential benefit of this combination remains speculative, as we are not aware of scientific evidence supporting its efficacy in this clinical setting [28].

### Ad 4: Timing and modality of aortic valve replacement in the context of antibiotic and surgical management of endocarditis

The indication for a surgical aortic valve replacement arised in cases of severe aortic valve insufficiency with cardiogenic shock following resuscitation. In an interdisciplinary consensus (heart team), a minimally invasive catheter-based procedure (TAVI) was considered the only possible treatment option as an off-label procedure, as the patient's general condition did not allow for conventional surgery. The plan here was to use TAVI to stabilize the circulatory situation to such an extent that further treatment of the necrotizing fasciitis was possible, and then to perform a secondary (mechanical) aortic valve replacement once the patient had stabilized.

The first TEE on day 2 showed a competent aortic valve with a large vegetation. The diagnosis of infective endocarditis was made, and a choice between conservative and surgical treatment had to be made. The role of surgery is (i) to control the infectious focus in the case of difficult-to-treat pathogens (e.g. by fungi or antibiotic-resistant bacteria) and/or deep-seated infection sites (e.g. prosthetic valve infection, presence of paravalvular abscesses) which are unlikely to be sterilized by antibiotics alone; (ii) to restore the structural and functional integrity of the valve, thus preventing or reversing heart failure; and (iii) to prevent potentially catastrophic systemic embolization events [29]. During the first days of the present case only (iii) preventing systemic embolization weighed in favour of early surgery because of the size of the vegetation, although mitral rather than aortic and staphylococcal or fungal pathogens rather than streptococci are believed to bear higher risks [29].

After an echocardiographic defect in the aortic valve with high-grade valve regurgitation was detected on day 12, we were confronted with the question of an adequate repair procedure.

A central consideration was the much-discussed question in literature about the timing of surgery when patients have severe valve dysfunction but do not have heart failure.

There are recommendations, to postpone surgery on the presumption that operating on a patient with active infection is too risky. The main challenge in this patient was that conventional surgical valve replacement was considered to carry prohibitive risk due to ongoing infection, recurrent hemorrhage, and multiorgan dysfunction [29, 30].

From day 12 onward, we followed the plan to rehabilitate the infectious situation of the stump to be able to perform an open aortic valve reconstruction. However, this original plan was gradually abandoned. For this open operative procedure, the intermittent use of a heart–lung machine with mandatory full heparinization would have been necessary. However, due to persistent haemorrhages from the gastrointestinal tract and recurrent haemorrhagic shock, surgical intervention was ruled out until the end because of high intraoperative bleeding risk.

Overall, the rate of early valve replacement or repair has increased over time in keeping with the prevailing opinion that surgery is a key component of the management of many complicated IE cases [3, 31]. Apart from the unfavourable bleeding situation in our patient, however, the evidence base for this practice is decidedly mixed. A single randomized trial by Kang et al. demonstrated a significant reduction in the composite outcome of inhospital death and embolic events with early surgery [32]. While clearly transformational, the generalizability of this study was nonetheless questioned. Study subjects were healthier and infected with less virulent pathogens (for example, Viridans streptococci) than our critically ill patient [3, 30].

Nevertheless, the pressure to treat the valve insufficiency increased day by day as progressive heart failure developed. Actually, the patient had an indication for surgery from day 12 on, because the principal consensus indications for valve surgery are heart failure, uncontrolled infection and prevention of embolic events in patients at high risk [3]. However, from days 12–22, our aim was to stabilize the patient sufficiently to allow for definitive surgical management.

As our patient was required resuscitation twice during surgical interventions on the stump, a TAVI was finally implanted as an emergency procedure on day 46.

There are only a few reports (first reported by Albu et al. in 2012) of aortic infective endocarditis (IE) treated with transcatheter aortic valve replacement (TAVR) [33–35].

However, since these few case reports describe a successful TAVI implantation with significantly higher freedom of heart failure in septic endocarditis, this procedure was certainly legitimised in this situation "with the back to the wall" [36, 37]. Nonetheless, a well-designed randomized prospective registry is urgently needed to investigate the outcomes of TAVI for this off-label indication [37].

Of course, the crucial question here was whether the infectious focus in the old aortic valve would heal well after TAVI despite all prolonged antibiotic therapy [36]. To the best of our knowledge, the anti-infective therapy we used at the time of TAVI implantation was targeted at all pathogens detected and was therefore intended to prevent re-infection of the fresh TAVI. According to García-Granja after 10 days of appropriate antibiotic treatment and in the absence of diabetes mellitus, Staphylococcus aureus, concomitant mitral involvement, or aortic prosthesis, the rate of positive cultures from excised valves is approx. 3.5 % [33].

However, TAVI was performed as an exceptional off-label hemodynamic rescue procedure rather than as definitive treatment of active infective endocarditis. Although TAVI resulted in an improvement in hemodynamics, it did not provide adequate infectious source control. The TAVI procedure was regarded initially as a bridge therapy to aortic valve surgical replacement in this patient, given the septic status and the prohibitive risk for classical surgery [33, 36]. The treatment strategy after TAVI implantation was focused on controlling the infectious process and achieving hemodynamic stabilization, with the ultimate goal of restoring the patient’s clinical condition sufficiently to allow subsequent definitive surgical aortic valve reconstruction.

One could discuss whether earlier treatment with TAVI would have improved the outcome. Since the operation would not have been possible for the foreseeable future, in retrospect an earlier TAVI would at least have slowed down progression of the heart failure. However, since VRE infestation of the new TAVI was detected post mortem, it is uncertain whether earlier implantation—possibly at a time with weaker antibiotic coverage—would really have been advantageous. Nevertheless, the implantation of the TAVI in our septic patient underlines the value of the integrative, multidisciplinary approach for complex clinical decisions and the real-life clinical challenges which sometimes require nonstandard, patient tailored therapeutical decisions [36].

### Ad 5: Possible dislocation or deformation of the TAVI due to resuscitation

Major complications following TAVI are considered to be mispositioning, valve migration/embolization, conversion to open surgery, need for pacemaker implantation, stroke, and myocardial infarction [38, 39]. Prosthesis dislocation during TAVI is a rare but serious complication, with an incidence ranging from 4 to 21 % [39].

Therefore, during resuscitation the clinicians considered dislocation of the aortic valve prosthesis and blocking the coronary arteries as the cause of the sudden circulatory failure. Echocardiography serves as the primary imaging modality for diagnosing complications post-TAVI, regardless of the timing of the disease [40]. In our case, however, the echocardiography performed intermittently during CPR showed no further movement of the heart. Moreover, we could not detect any peri-annular complications.

In cases of peri-annular complications, the sensitivity of TTE diminishes to 36–69 %, while TEE maintains a higher sensitivity, reported between 86 and 100 % [40]. Nevertheless, using TTE and TEE to detect small vegetations or paravalvular abscesses is challenging due to the shadowing effect and reflectance of prosthesis material. Moreover, detecting incipient vegetation in the free space between the transcatheter and native aortic valve, amidst extensive calcifications and prosthetic material, is particularly challenging using TTE and TEE, if even possible [40, 41].

TTE and TEE may also be unable to differentiate between vegetations and fibrous strands or thrombi [40, 42].

Despite these challenges, while TTE is recommended as the initial imaging procedure for all patients suspected of having prosthetic valve infection, it does not hold the status of the gold standard [40, 43].

Another possible imaging modality that might have assisted in diagnosing peri-annular complications after TAVI might have been contrast-enhanced cardiac computed tomography angiography (CCTA) [39, 40].

As in our case, the clinical assessment, laboratory findings, and first-line imaging modalities such as echocardiography may raise suspicion but often fail to reliably define the extent and clinical relevance of complications. CT, however, allows for a more precise identification of the underlying cause [39].

As no complications could be identified by echocardiography, CCTA could have provided an alternative. However, given the patient’s critical condition and the need for ongoing CPR, transfer to the CT scanner was not feasible, and CCTA was therefore not an option. Moreover, current evidence does not support the routine use of CT after TAVI, as a prognostic benefit has not yet been demonstrated [39, 40].

The clinicians also interpreted the occurrence of pacemaker dependency without any sufficient ventricular rhythm about a week after the TAVI procedure as sign of a potentially existing paravalvular abscess. About 11–15 % of patients after TAVI procedure are in need of a permanent pacemaker [44]. Mostly the pacemaker dependency after TAVI procedure was associated with the first postprocedural rhythm [44]. Although the first postprocedural rhythm of our patient was sinus rhythm, he developed a heart block with secondary pacemaker dependency. Therefore, it is important that nearly 40 % of patients with infective endocarditis are affected by perivalvular abscesses. The location of the atrioventricular node is near the aortic valve which was severely affected by endocarditis in our patient. Abscesses in this area are often associated with heart blocks which can lead in pacemaker dependency [45].

Therefore, delayed atrioventricular block after TAVI is not uncommon, and temporary pacing is routinely maintained for several days because mechanical interaction of the prosthetic valve with the conduction system may result in transient or delayed conduction disturbances [45]. In our patient, however, a new high-grade AV block occurred approximately one week after the procedure and ultimately required permanent pacemaker implantation.

This finding might have raised a stronger suspicion for periannular extension of infection and prompted repeat imaging, particularly repeat TEE, to evaluate for the presence of a paravalvular abscess.

Later the autopsy revealed a large paravalvular abscess in our patient. On retrospective review, neither the pre-procedural cardiac CT nor TEE showed evidence suggestive of peri-annular abscess formation; The delayed AV block, as a new conduction abnormality, in the setting of aortic valve endocarditis, should be considered highly suggestive of periannular infection, until proven otherwise. We acknowledge that a post-TAVI cardiac CT or TEE (both were not performed) might have facilitated earlier detection of evolving peri-annular complications.

The need for post-interventional imaging following TAVI performed as a rescue strategy represents an important educational aspect of this case, as early imaging may facilitate the detection of evolving peri-annular complications [39, 40].

### Ad 6: ECLS in sepsis – a feasible option for our patient?

We could also discuss whether VA-ECMO during severe cardiogenic shock, instead of TAVI implantation, had a better chance of survival. There is no evidence of improvement of the clinical outcomes in patients with rapidly deteriorating or severe cardiogenic shock with immediate implantation of VA-ECMO compared to conservative strategy [46]. There was also a high risk of bleeding as therapeutic anticoagulation is needed to guarantee function of the ECMO device.

Implantation of VA-ECMO in patients with severe sepsis-induced cardiogenic shock leads to improved short-term survival rates [47]. Patients must be selected carefully because this salvage therapy only improved survival in patients suffering concomitant myocardial depression with persistently low cardiac output. In patients without severe myocardial depression in septic shock VA-ECMO is associated with poor outcome [48]. Furthermore, this consideration must be viewed from a different perspective in patients with severe aortic regurgitation. In this setting, peripheral VA-ECMO may increase left ventricular afterload and thereby aggravate aortic regurgitation, potentially leading to left ventricular distension and pulmonary edema. Therefore, the use of VA-ECMO in severe aortic regurgitation requires careful consideration of the potential hemodynamic consequences.

ECLS, as VA-ECMO, during resuscitation showed an increased short-term and long-term survival with better neurological outcomes than CPR alone [49, 50]. Therefore, we discussed the implantation of VA-ECMO during the last resuscitation of our patient. At this time the infection in his left femur was still active and we suspected spreading in his pelvis and abdominal area. Chirurgical options here were limited. Given the active infection, recurrent major gastrointestinal bleeding, persistent severe heart failure and prolonged organ dysfunction, there was no realistic bridge to definitive surgical treatment or feasible exit strategy at that time. In interdisciplinary consensus we refrained from VA-ECMO implantation.

Because of the sudden circulatory failure, the clinicians considered dislocation of the aortic valve prosthesis and blocking of the coronary arteries. Life threatening complications after TAVI – Implantation remain rare. About 0,7–1 % patients are in need of emergency cardiac surgery after TAVI-Implantation, most common due to left ventricular perforation (28.3 %) and annular rupture (21.2 %). Less frequent are by valve embolization/migration (12.7 %) and coronary obstruction/occlusion (5.2 %). Those patients who were affected by complications suffered a high in-hospital mortality [51]. Most common complications occur during TAVI-procedure or in in the first 7 days after implantation [52].

## Conclusion and take home messages

- Complex septic patients require multidisciplinary decision-making.
- Early radical surgery saves lives.
- Temporary loop colostomy may provide meaningful source control.
- Combination of proton pump inhibitor and misoprostol in refractory gastrointestinal bleeding may be beneficial.
- Paravalvular abscesses may remain clinically occult.
- The optimal timing and role of VA-ECMO in severe cardiogenic shock secondary to infective endocarditis remain uncertain.
- TAVI represent even in endocarditis a last-resort rescue option for uncontrolled heart failure.
- Early post-interventional imaging after rescue TAVI is crucial for detecting evolving peri-annular complications.

## Supplementary Information

Supplementary Material 1: Video Descriptions

Supplementary Material 2: Video 1

Supplementary Material 3: Video 2

Supplementary Material 4: Video 3

Supplementary Material 5: Video 4

## Data Availability

No datasets were generated or analysed during the current study.
