# Supplementary material for: Multiorgan failure in a young patient with necrotizing fasciitis: a case report of septic shock progressing to mixed shock with fatal cardiac outcome
Source: J Med Case Rep. 2026 Nov 13;20:742. doi: 10.1186/s13256-026-06805-2 (PMC13760787; doi:10.1186/s13256-026-06805-2)
Supplement: Supplementary file 1 [file 13256_2026_6805_MOESM1_ESM.docx]

# Supplementary Video Descriptions

**Video 1:**

**Transesophageal echocardiographic assessment of the native aortic valve on day 2 demonstrated a highly mobile vegetation attached to the native aortic valve**

**Sequence 1:** Mid-esophageal long-axis view at 127°

**Sequence 2:** Zoomed mid-esophageal long-axis view at 127°

**Sequence 3:** Mid-esophageal short-axis view at 82°

**Sequence 4:** Mid-esophageal view at 10°

**Video 2:**

**Transesophageal echocardiographic assessment of the native aortic valve on day 15 demonstrated aortic regurgitation and aortic regurgitant jet on color Doppler.**

**Sequence 1:** Mid-esophageal long-axis view at 124°

**Sequence 2:** Mid-esophageal short-axis view at 60°

**Sequence 3:** Mid-esophageal long-axis view at 123°

**Video 3:**

**Follow-up transesophageal echocardiographic assessment of the native aortic valve on day 38 demonstrated further valve damage and increasing aortic regurgitation on color Doppler**

**Sequence 1: Mid-esophageal long-axis 2D view at 120°**

**Sequence 2: Mid-esophageal long-axis view at 123° with color Doppler**

**Sequence 3: Mid-esophageal short-axis 2D view at 61°**

**Sequence 4: Mid-esophageal short-axis view at 61° with color Doppler**

**Video 4. Sequential implantation of two 29-mm Edwards SAPIEN 3 transcatheter heart valves**

**Sequence 1 – Implantation of the first SAPIEN 3 valve**
Fluoroscopic imaging demonstrating positioning and deployment of the first 29-mm Edwards SAPIEN 3 transcatheter heart valve.

**Sequence 2 – Implantation of the second SAPIEN 3 valve**
Fluoroscopic imaging demonstrating positioning and deployment of a second 29-mm Edwards SAPIEN 3 transcatheter heart valve within the first prosthesis.

# Detailed Description of the TAVI Implantation Procedure

According to our institution’s protocol, a transjugular pacemaker was inserted for rapid pacing during the implantation and for the case of postinterventional atrioventricular blockade.

After cannulation of the femoral artery a 6-French sheath was introduced into the left common femoral artery and a 5 Fr sheath into the left femoral vein. Via crossover technique from the left-sided arterial access, angiography of the right iliac axis was performed to guide fluoroscopy-assisted puncture of the right common femoral artery, into which an arterial sheath was placed. Heparinization with an ACT of >200 s was performed.

Then, a pigtail catheter was advanced via the transfemoral 6-French sheath and positioned in the non-coronary cusp of the aortic valve. Bulbography was performed to verify the implantation plane as preoperatively determined by CT; however, due to severe aortic insufficiency, contrast delineation was limited. No mechanical resistance was encountered at the annular level because of a flail leaflet, precluding the placement of a marking wire. Simultaneous hemodynamic recordings from the left ventricle and ascending aorta confirmed complete aortic valve incompetence, and transesophageal echocardiography (TEE) revealed a previously known, freely mobile valve leaflet.

A 29 mm Edwards Sapien 3 transcatheter heart valve, selected based on pre-procedural CT assessment, was implanted. Initial TEE confirmed good valve function; however, early hemodynamic assessment revealed migration of the prosthesis approximately 1 cm toward the LVOT with resulting supraskirtal aortic insufficiency, necessitating implantation of a second 29 mm Sapien 3 valve (same type) following re-crossing of the native valve.

The evaluation of valve function, now performed using transesophageal echocardiography (TEE), invasive hemodynamic measurement, and bulbography, showed normal opening characteristics of the prosthesis.
